# Murine Model of Venous Bypass Grafting to Study Determinants of Graft Patency

**DOI:** 10.1002/cpz1.70466

**Published:** 2026-09-23

**Authors:** Vanessa Heim, Maria Ioannou‐Nikolaidou, Anh‐Vu Nguyen, Lynn Muller, Veronika Niedrist, Jakob Hirsch, Jonas Eder, Dominik Hau, Leo Winter‐Pölzl, Clemens Engler, Ronja Lohmann, Felix Nägele, Gerald Brandacher, Stefan Schneeberger, Nikolaos Bonaros, Michael Grimm, Can Gollmann‐Tepeköylü, Michael Graber

**Affiliations:** ^1^ Department of Cardiac Surgery Medical University Innsbruck Innsbruck Austria; ^2^ Institute of Clinical and Functional Anatomy Medical University Innsbruck Innsbruck Austria; ^3^ Daniel Swarovski Transplantation Research Laboratory, Department of Visceral, Transplant and Thoracic Surgery Medical University of Innsbruck Innsbruck Austria; ^4^ Department of Thoracic Surgery University Hospital Zurich Zurich Switzerland

**Keywords:** graft patency, laser Doppler imaging, mouse model, step‐by‐step video, venous bypass grafting

## Abstract

The investigation of patency and remodeling in venous grafts requires a reproducible animal model that enables the study of the underlying cellular and molecular processes. Here, we describe a simplified mouse model for venous bypass grafts, in which the inferior thoracic vena cava (IVC) of a donor mouse is interposed into the common carotid artery of a recipient mouse using a cuff technique. The procedure is divided into four basic protocols, which includes the harvesting of the inferior vena cava from donor mice (Basic Protocol 1), the preparation of the common carotid artery (Basic Protocol 2), the interposition of the inferior vena cava graft (Basic Protocol 3), and post‐operative care (Basic Protocol 4). Detailed step‐by‐step instructions and an instructional video are provided to facilitate microsurgical training and to improve the reproducibility and accessibility of the procedure. This model enables the investigation of the patency and remodeling of venous grafts and provides a platform for researching the cellular and molecular mechanisms contributing to venous graft failure, as well as for evaluating potential therapeutic interventions. © 2026 The Author(s). *Current Protocols* published by Wiley Periodicals LLC.

**Basic Protocol 1**: Harvest of inferior caval vein from donor mice

**Basic Protocol 2**: Preparation of the common carotid artery

**Basic Protocol 3**: Inferior vena cava graft interposition

**Basic Protocol 4**: Postoperative management

## INTRODUCTION

Coronary artery disease represents the leading cause of death in the western world. In patients with left main or multivessel coronary artery disease, coronary artery bypass grafting (CABG) continues to be recommended by contemporary clinical guidelines (Neumann et al., 2019). It remains the most common performed cardiac surgical intervention worldwide (Lawton et al., 2022; Melly et al., 2018). In CABG surgery, autologous venous and arterial grafts are used to circumvent stenotic coronary arteries, thereby restoring myocardial perfusion and preventing progressive myocardial damage (Gerodias et al., 2025). The left internal thoracic artery is considered the gold‐standard conduit for coronary artery bypass grafting, with reported 10‐year patency rates of 85% to 95%. Other conduit options include the radial artery, which generally has superior long‐term patency to conventional saphenous vein grafts and may achieve 10‐year patency rates of 80% to 90% when grafted to appropriately selected, severely stenosed coronary targets. Saphenous vein grafts are less durable, with 50% to 60% remaining patent at 10 years (Desai et al., 2004; Goldman et al., 2004). Venous graft failure is a multifactorial process that develops over time and can be divided into distinct stages. Early graft failure is mainly caused by acute thrombosis, while intimal hyperplasia contributes to progressive narrowing of the graft lumen during the mid‐term period. In the later stages, accelerated atherosclerosis can lead to graft failure and occlusion. Although intimal hyperplasia plays an important role in venous graft failure, the molecular and cellular mechanisms underlying its development are not fully understood. Therefore, effective strategies to prevent or reduce intimal hyperplasia and improve long‐term graft patency remain limited (de Vries et al., 2016; Guida & Angelini, 2022).

Investigating the patency of arterial and venous grafts requires an animal model that allows the analysis of multiple contributing factors. The use of the thoracic inferior vena cava (IVC) as a graft is an established small‐animal model for studying venous graft patency and remodeling (Yu et al., 2010; Zou et al., 1998). However, existing small‐animal protocols are often technically demanding and complex. Here, we present a simplified mouse model accompanied by step‐by‐step instructional videos to make the procedure more accessible and reproducible. This model enables the investigation of venous graft patency and remodeling, as well as the factors influencing graft patency.

To better illustrate the methodological advances of the present model compared with previously published venous graft models, we compared our approach with established models reported by Zou et al. (1998) and Schleimer et al. (2012). Zou et al. (1998) described a microsurgical mouse model of venous bypass graft arteriosclerosis, whereas Schleimer et al. (2012) established a rat vein interposition model and provided a step‐by‐step instructional video to facilitate microsurgical training. In comparison with these established approaches, the present article combines a simplified surgical procedure with detailed step‐by‐step instructions and an accompanying instructional video specifically adapted to the mouse model. Table 1 provides a comparison of previously established venous graft models to the model presented in this article.

**Table 1:** Comparison of Previously Established Venous Graft Models

Parameter	Zou et al. (1998)	Schleimer et al. (2012)	Present article
Species	Mouse	Rat	Mouse
Model	Venous bypass graft model	Vein interposition model	Mouse vein graft model
Primary application	Graft arteriosclerosis	Bypass graft patency	Study of venous graft remodeling and patency
Graft source	Venous graft	Venous graft	Venous graft
Recipient vessel	Arterial vessel	Arterial vessel	Arterial vessel
Surgical technique	Microsurgical vein grafting	Vein interposition	Standardized microsurgical grafting technique
Detailed step‐by‐step protocol	No	Yes	Yes
Instructional video	No	Yes	Yes
Operative time	60 min	65 min	∼10 min (Basic Protocol 1); ∼50 min (Basic Protocols 2 and 3)
Operative success rate	90%	Not reported	100%
Postoperative survival	94.74 %	100 %	100% at 4 weeks
Graft patency	100% at harvest time	71% at harvest time	100% at day 28
Patency assessment	Histological assessment of grafts	Doppler principle	Laser Doppler imaging
Learning curve	Technically simple; easy to learn; cuff model	Gain expertise quickly; step‐by‐step video; cuff model	Easy to learn; technically simple; gain expertise quickly; step‐by‐step video; cuff model
Main methodological advantage	Established mouse model for graft arteriosclerosis	Video‐supported vein interposition model in rats	Video‐guided, standardized mouse IVC graft model

### Surgical success rate

Zou et al. (1998) reported a surgical success rate of ∼90%, while Schleimer et al. (2012) did not report a surgical success rate. In the present model, graft implantation was successfully performed in all animals included in the study, resulting in a surgical success rate of 100%. No postoperative mortality was observed, corresponding to a postoperative survival rate of 100% at 4 weeks. Thus, the present model demonstrates a high degree of surgical feasibility and reproducibility.

### Duration of surgery

The surgical procedure required ∼60 min in the model described by Zou et al. (1998) and ∼65 min in the model of Schleimer et al. (2012). In comparison, the present protocol requires ∼10 min for Basic Protocol 1, while Basic Protocols 2 and 3 together require ∼50 min when performed by microsurgeons with prior experience. Although direct comparison is limited by differences in the definition and reporting of operative time, the present article provides a relatively time‐efficient approach to establishing the mouse venous graft model.

### Learning curve for inexperienced microsurgeons

Zou et al. (1998) described their cuff‐based model as technically simple and easy to learn, while Schleimer et al. (2012) demonstrated that microsurgical expertise can be acquired rapidly using a cuff‐based technique supported by step‐by‐step video guidance. Similarly, the present article combines a technically simple cuff‐based approach with detailed step‐by‐step instructions and an instructional video. Based on our experience, the technique is easy to learn and allows rapid acquisition of microsurgical expertise, depending on the operator's prior experience. Although no formal learning‐curve analysis was performed, these features are intended to facilitate technique acquisition, standardization, and reproducibility.

### Patency rate

Zou et al. (1998) reported a graft patency rate of 100% at the time of graft harvest, while Schleimer et al. (2012) reported an overall patency rate of 71% at harvest. In the present model, laser Doppler imaging demonstrated preserved blood flow through the implanted graft in all animals at day 28, corresponding to a graft patency rate of 100%. Throughout the 4‐week follow‐up period, all animals maintained normal food and water intake and social behavior, with no observed wound infections, neurological deficits, impaired drinking, or stroke‐like symptoms.

Taken together, the present model combines a high surgical success rate and graft patency with a comparatively short procedure time and a standardized, video‐guided training approach. In contrast to the previously described models, the present article provides a detailed and reproducible method specifically adapted to mice using the thoracic inferior vena cava as the venous graft source. These features may facilitate the acquisition and standardization of microsurgical venous grafting techniques and support broader adoption of the model for studies of venous graft remodeling and patency.

## STRATEGIC PLANNING

Ethics committee approval was obtained for all animal experiments (GZ 2025‐0.281.950). C57BL/6JRj mice of both sexes were used as donors and recipients for vein grafts, which were purchased at the age of 12 weeks from Janvier Labs (RRID:IMSR_RJ:C57BL‐6JRJ). All animals were acclimated for 1 week on a light/dark (12 hr/12 hr) cycle at 24°C and received food and water ad libidum prior to experimentation.

*NOTE*: All protocols involving animals must be reviewed and approved by the appropriate Animal Care and Use Committee and must follow regulations for the care and use of laboratory animals.

## Basic Protocol 1 HARVEST OF INFERIOR CAVAL VEIN FROM DONOR MICE

This protocol describes the surgical procedure for harvesting the thoracic inferior vena cava from a genetically identical donor mouse. The objective is to obtain the longest possible segment of the thoracic inferior vena cava with minimal residual adventitial tissue, as a part of the graft length will be lost during the procedure described in Basic Protocol 3. This surgical procedure can be completed within ∼10 min.

### Materials

C57BL/6JRj 12‐week‐old mice of both sexes (Janvier Labs, RRID:IMSR_RJ:C57BL‐6JRJ)
Surgical anesthetics/analgesics:
  Isoflurane (Zoetis, cat. no. TU061220)
  Medetomidine (100 mg/ml) (Domitor, Orion, cat. no. 8‐00144)
  Midazolam (5 mg/ml) (Midazolam Accord, Accord Healthcare, cat. no. 00065896)
  Fentanyl (0.05 mg/ml) (Fentanyl‐Hameln, Hamel Pharma, cat. no. 06143410)
  Atipamezole (5 mg/kg) (Antisedan, Orion, cat. no. 8‐00145)
  Flumazenil (0.1 mg/kg) (Anexate, Cheplapharm Arzneimittel, cat. no. 1‐18341)
  Naloxone (0.4 mg/kg) (NALOXon, B. Braun, cat. no. 2240200)
  NaCl (0.9%), physiological saline solution (Current Protocols, 2006)
  Buprenorphine (0.3 mg/ml) (Bupaq, VetViva, cat. no. 0022‐2142)
  Tramadol (100 mg/ml) (Tramabene, TEVA B.V., cat. no.1307723)
  Heparin (130 I.E intraperitoneal for full heparinization of the mice) (Heparin Gilvasan, Gilvasan Pharma, cat. no. 3909981)
  Heparin (100 IU/ml for flushing) (Heparin Gilvasan, Gilvasan Pharma, cat. no. 3909981)
  Optixcare ophthalmic lubricant (Aventix Animal Health Corp, cat. no. OPX‐4242)

Surgical supplies:
  Polyethylene cuff, 0.37‐mm inner diameter 27G (MicroLumen, cat. no. 145)
  Scissors (FST, cat. no. 14058‐11)
  Micro forceps (Scanlan, cat. no. 4004‐280)
  Micro spring handle scissors (AROSurgical, cat. no. 11.602.11)
  Micro needle holder (DiaDust, B. Braun, cat. no. FM540R)
  S&T vessel dilator (S&T, cat. nos. D‐5a.2 and D‐5a.1))
  Aneurysm clips (B. Braun, cat. no. FT260T)
  Aneurysm clip applier (Aesculap, B. Braun, cat. no. FE570K)
  8‐0 silk sutures (Pearsalls, cat. no. 55A04200BLACK)
  4‐0 polypropylene suture, non‐resorbable (Ethicon, cat. no. EH7774H)
  3‐0 Vicryl rapid suture (Ethicon, cat. no. V115)

#### Anesthesia

All recipient mice were anesthetized by an intraperitoneal injection of a combination of Medetomidine (500 µg/kg), Midazolam (5 mg/kg), Fentanyl (50 µg/kg), and NaCl (0.9%). Ophthalmic ointment will be applied to both eyes to prevent corneal drying during the procedure. Afterwards a reversal agent Atipamezole (5 mg/kg), Flumazenil (1 mg/kg), Naloxone (2.4 mg/kg) and NaCl (0.9%) was administered. All donor mice are euthanized by administering an overdose of isoflurane (100%) until deep anesthesia and death occur. Cervical dislocation is then performed to confirm death.

#### Cuff preparation

**Figure 1:**
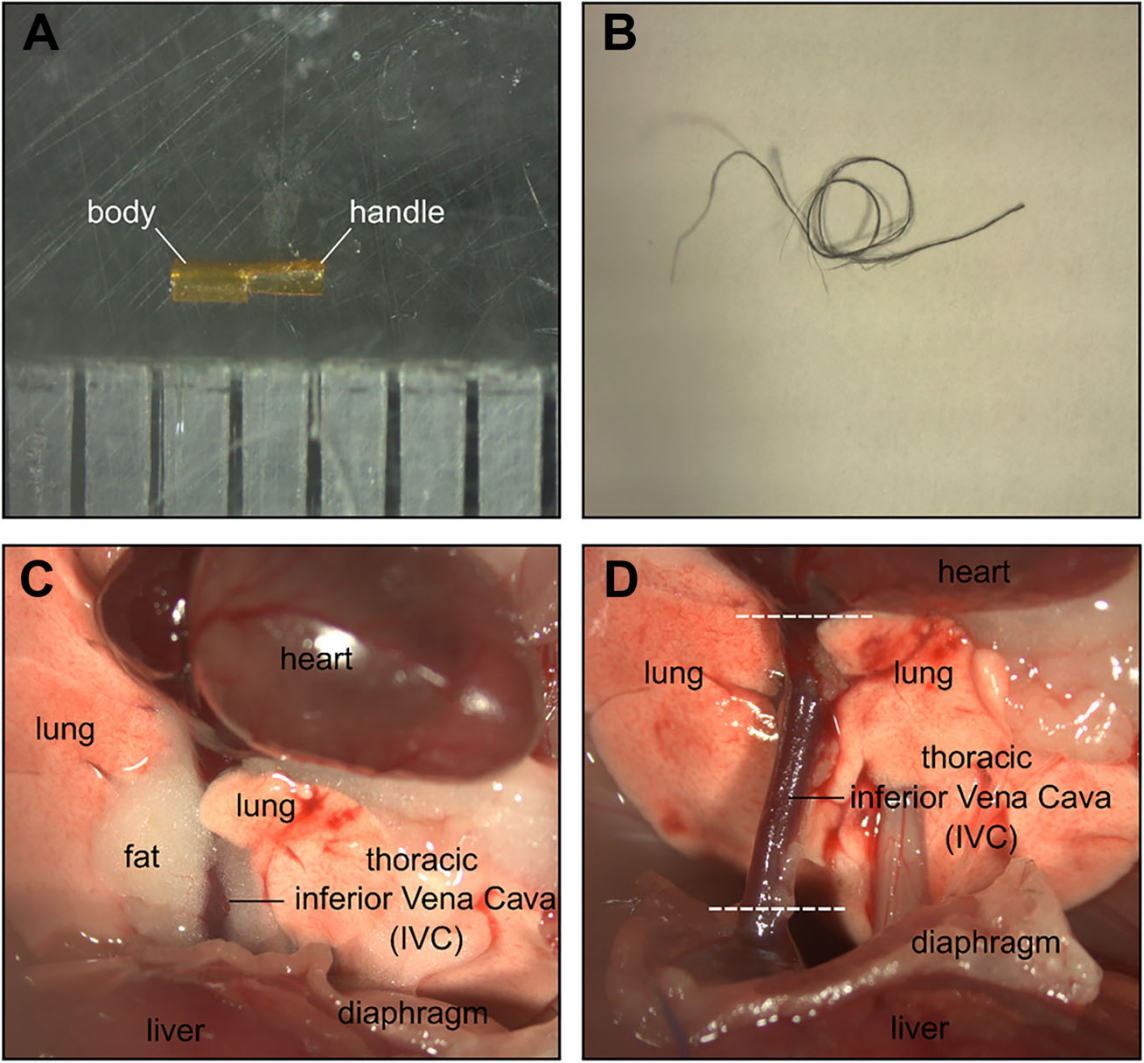
Operative Preparation. (**A**) 2‐mm cuff, 1‐mm cuff body, 1‐mm cuff handle. (**B**) 8‐0 silk suture double ligation loop. (**C**) Inferior vena cava in situ. (**D**) Inferior vena cava cleaned; mark where to cut (white).

A polyethylene cuff was used. The cuff was cut in the length of 2 mm by hand. The cuff body and the handle should be equally long (Fig. 1A). For the procedure, four double‐loop 8‐0 silk sutures were also prepared and set aside for use during the graft anastomosis (Fig. 1B).

#### Surgical procedure

1 Anesthetize donor mice with 100% isoflurane and then euthanize by cervical dislocation (see above).
2 For vena cava preparation, perform a median laparosternotomy and bilateral thoracotomy in the donor animal using surgical tools.
3 First, carefully remove the fat surrounding the thoracic inferior caval vein to fully expose the vessel (Fig. 1C‐D).
  Be very careful, as the IVC is very thin‐walled and ruptures easily when damaged.
4 Next, harvest the IVC from the right atrium down to the diaphragm under sterile conditions and place it in a heparin solution with no prior flushing necessary (Fig. 1D).

## Basic Protocol 2 PREPARATION OF THE COMMON CAROTID ARTERY

This protocol presents the preparation of the recipient common carotid artery using the cuff technique. The artery is carefully isolated, mounted onto cuffs at both ends, everted, and secured with ligatures to create a stable anastomotic site while maintaining vessel integrity and patency. This surgical procedure can be completed within ∼30 min.

### Additional Materials (also see Basic Protocol 1)

Cotton‐tipped swaps

#### Surgical procedure

**Figure 2:**
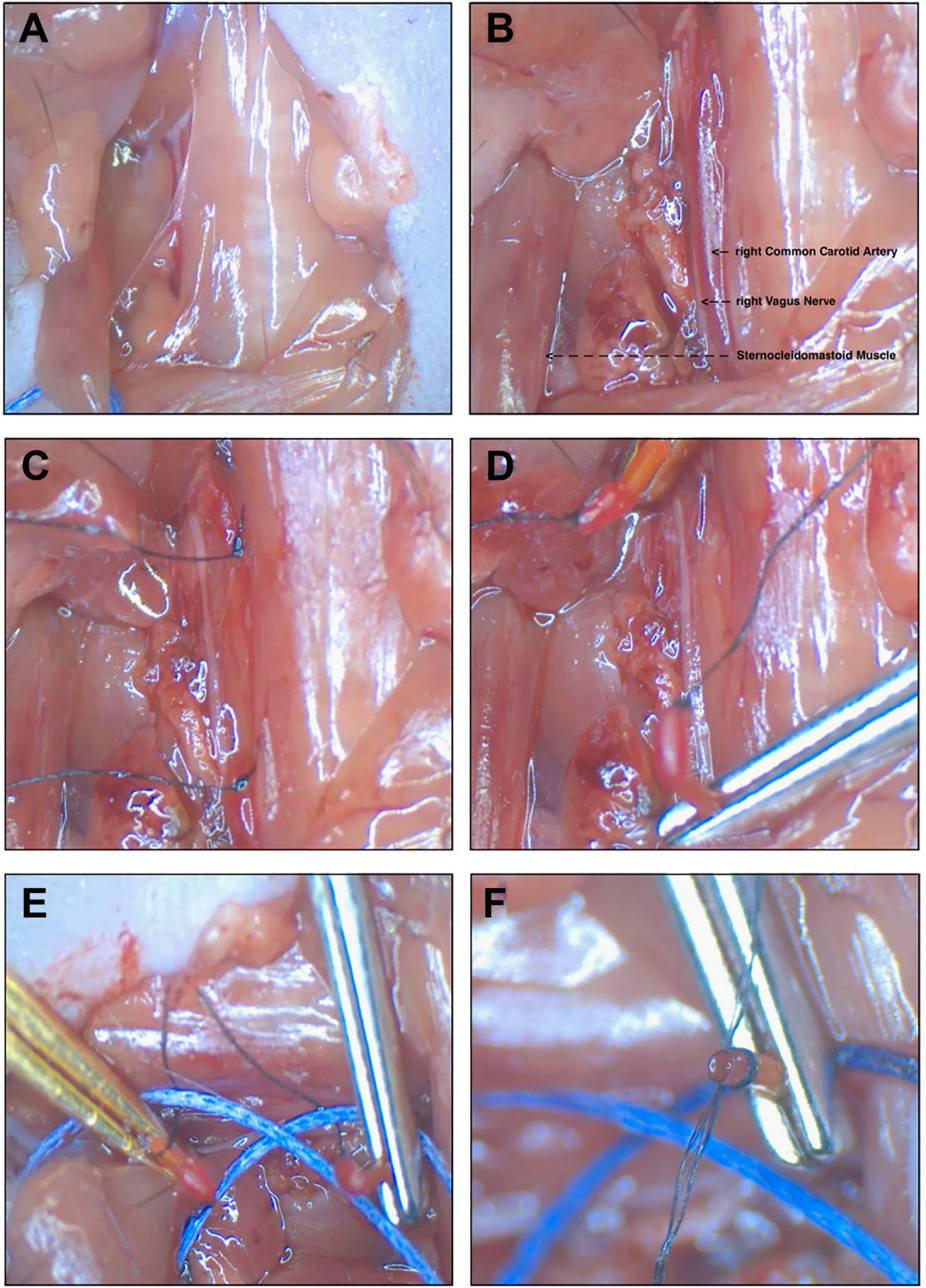
Preparation of the common carotid artery (CCA). (**A**) Mobilized sternocleidomastoid muscle. (**B**) Mobilized common carotid artery (CCA), Vagus nerve. (**C**) Ligated CCA. (**D**) Cuffs slipped over the distal ends of the CCA, clipped cuff handle and artery. (**E**) 4‐0 Vicryl suture wrapped around both clips. (**F**) Inverted CCA secured with 8‐0 double loop ligation.

1 Anaesthetize recipient mice with intraperitoneal anesthesia as described in Basic Protocol 1.
2 Perform a right median cervical incision from the manubrium sterni to the ear using surgical tools.
3 Carefully mobilize the sternocleidomastoid muscle, lift the muscle gently using a 4‐0 Prolene suture for better exposure of the underlying structures (Fig. 2A; Video 1, minute 00:15–00:20; see Supporting Information for a transcription of the video).

**Video 1:** Instructional video of venous bypass graft model in mice. The video demonstrates the surgical procedure for creating a venous bypass graft model in mice. In the recipient mouse, the common carotid artery is exposed and temporarily clamped. The donor vein is then inserted into the carotid artery using a cuff technique. The graft is secured at the arterial ends, the clamps are released, and the patency of the graft and blood flow are assessed visually. This video is intended to facilitate microsurgical training and improve the reproducibility of the procedure.

4 Remove the internal jugular vein around the common carotid artery and mobilize the vagus nerve.
  Make sure to not compress the artery and nerve.
5 Mobilize the common carotid artery from the bifurcation down to the brachiocephalic trunk (Fig. 2B; Video 1 minute 00:30–00:37).
6 Ligate the artery at two points with an 8‐0 silk suture while carefully avoiding injury to the nerve before transecting the artery (Fig. 2C; Video 1, minute 00:45–01:22).
  Use silk sutures instead of Prolene, as Prolene is too stiff and tends to slip.
7 Place the cuff over the caudal end of the artery using the long silk suture to guide it in position (Video 1, minute 01:32–01:44).
8 Clamp the artery and the cuff exactly on the handle with an aneurysm clip as far distally as possible to secure the vessel and cuff in place (Fig. 2D; Video 1, minute 01:48–01:53).
  It is very important that the artery and cuff handle are clamped with the clip, otherwise the stump of the artery will slip into the cuff.
9 Perform the same procedure on the cranial end of the artery. When done, use a 3‐0 Vicryl suture and carefully wrap in around the clips (Fig. 2E; Video 1, minute 01:57–02:07).
10 Cut the caudal artery directly behind the ligature (the arterial stump should be equally long as the cuff body) and rinse the lumen with 100 IU/ml heparin solution (Video 1, minute 02:10–02:29).
  It is important to flush the stump with heparin to prevent blood clots.
11 Take the stump of the artery and carefully evert it over the cuff body, use a cotton swab to hold the stump in position (Video 1, minute 02:44–03:10).
12 Pull the double ligation loop around the everted artery and cuff, tie it tightly, and secure with a third knot (Fig. 2F; Video 1, minute 03:12–03:48).
13 Rinse again with heparin solution.
14 Perform the same procedure with the cranial arterial stump.

## Basic Protocol 3 INFERIOR VENA CAVA GRAFT INTERPOSITION

This protocol outlines the transplantation of a harvested venous graft into the prepared carotid artery using the cuff technique. After the graft has been positioned and secured, systemic heparinization is performed before restoring blood flow. Successful implantation is verified by visible pulsation and graft perfusion prior to wound closure. This surgical procedure can be completed within ∼20 min.

### Additional Materials (also see Basic Protocol 1)

Adhesive tape
Cotton‐tipped swabs
Laser Doppler imager (Moor instruments, cat. no. moorLDI2‐IR)

#### Surgical procedure

**Figure 3:**
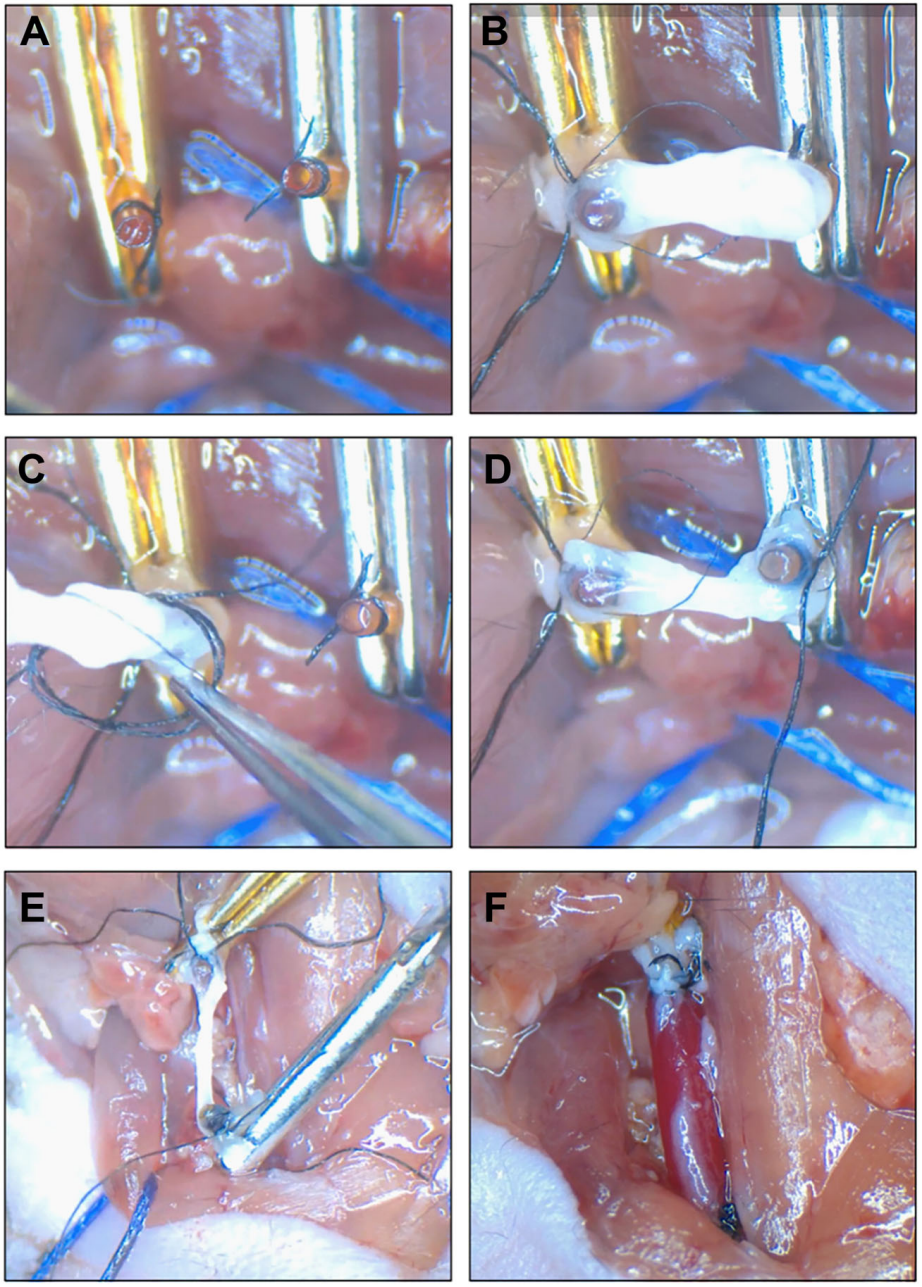
Inferior vena cava (IVC) interposition. (**A**) Approximation of both stumps. (**B**) Interposition of the IVC on the cranial stump with 8‐0 double loop ligation. (**C**) Position of the next double loop. (**D**) Interposition of the IVC. (**E**) Interposition of IVC without temporary sutures. (**F**) Reperfusion of the IVC interposition graft.

1 Use the already placed 3‐0 Vicryl suture around the clips and crisscross and pull the sutures to bring the two ends of the arteries closer together. Secure with adhesive tape. (Fig. 3A; Video 1, minute 04:18–04:29).
  Do not tighten too much, otherwise the carotid artery will be damaged.

2 Place the vein segment beside the artery stumps.
3 Rinse the cranial stump again with a heparin solution before placing the vein.
4 Position the venous end from the diaphragmatic side over the cranial arterial stump (Video 1, minute 04:49–05:05).
  Use the diaphragmatic end first, as it is narrower and more difficult to evert over the cuff. Once the vein is secured, the wider cardiac end can be more easily positioned over the second cuff.
5 Pull the double ligation loop around and knot it tightly, secure with a third knot, ensure that the vein is completely and evenly tightened (Fig. 3B; Video 1, minute 05:07–05:42).
6 Position the last double ligation loop around the vein (Fig. 3C; Video 1, minute 05:45–05:50).
7 Rinse the caudal arterial stump with heparin solution and pull the vein over, use a cotton swab to hold the vein in position (Video 1, minute 05:54–06:18).
8 Pull the last double loop ligation over the vein and secure with a third knot (Fig. 3D‐E; Video 1, minute 06:20–06:44).
9 Complete full systemic heparinization (130 IU heparin) via IP injection before opening the clips.
10 After 2 min remove the cranial clip first, then the caudal one (Video 1, minute 07:00–07:23).
11 Pulsation and blood flow can be seen if the surgery was successful (Fig. 3F; Video 1, minute 07:32–07:45).
12 Remove all temporary sutures that were used to hold structures in place.
13 Close the incision with a running suture using 4‐0 Prolene.
14 Perform Laser Doppler imaging.

## Basic Protocol 4 POSTOPERATIVE MANAGEMENT

This protocol provides an overview of the postoperative care procedures and monitoring of mice after surgery. During recovery, animals are maintained under warm conditions, closely observed, and treated with appropriate analgesics to minimize pain and support animal welfare.

### Additional Materials (also see Basic Protocol 1)

Infrared lamp (Beurer, cat. no. RA2952549)
Heat mat (Giangarden, cat. no. 37125)

1 Keep all mice warm under a heat lamp and on a heat mat during recovery.
2 Check the mice every 20 min over a period of 2 hr. Check daily; if mice are in pain, inject subcutaneous buprenorphine injections (0.03 to 0.05 mg/kg bodyweight), if necessary.
3 Provide analgesia with subcutaneous buprenorphine injections (0.03–0.05 mg/kg bodyweight) and add 0.2 mg/ml tramadol to the animals’ drinking water as a precautionary pain therapy.

## COMMENTARY

### Representative Results

Daily postoperative monitoring revealed no adverse events, including wound infection, neurological deficits, impaired drinking, or stroke‐like symptoms. All mice drank and ate normally throughout the experiment and behaved socially. A postoperative survival rate of 100% was observed during the 4‐week follow‐up period.

**Figure 4:**
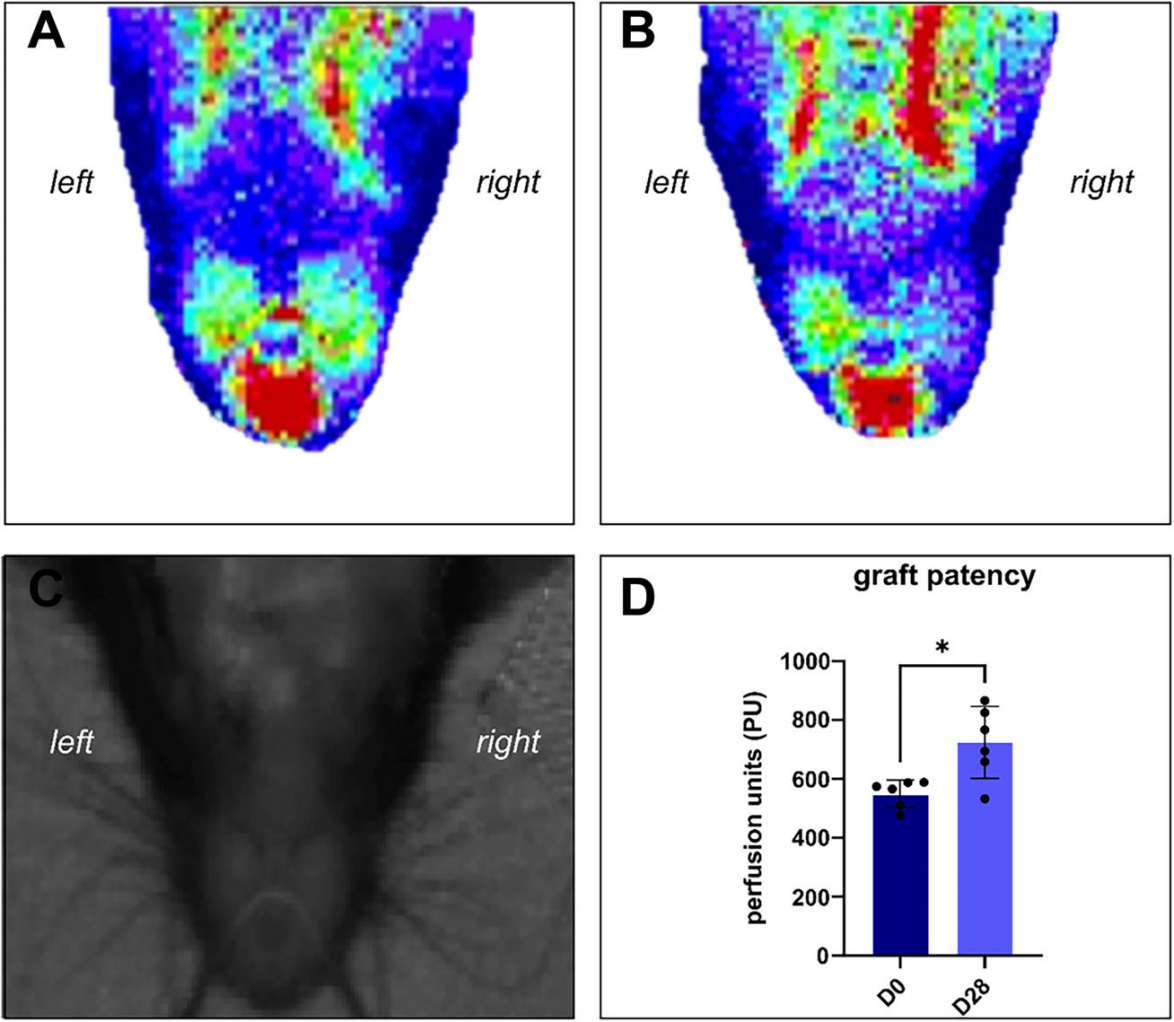
Laser Doppler images. (**A**) Laser Doppler before operation. (**B**) Laser Doppler before harvest. (**C**) Illustration of mouse during laser Doppler. (**D**) Perfusion units (PU) on the operated side increased significantly from day 0 (D0) to day 28 (D28), indicating graft patency. Data are presented as mean ± *SD*; *p* < .05.

During the follow‐up period, blood flow through the implanted graft was analyzed using laser Doppler imaging. As illustrated in Figure 4A‐D, right‐sided perfusion increased in all animals at pre‐harvest (D28), with no evidence of reduction, confirming patency of all vessels.

### Critical Parameters

#### Basic Protocol 1: Harvest of inferior caval vein from donor mice

A critical element for successful vena cava harvesting is the clear and careful exposure of the inferior vena cava. Adequate removal of surrounding adipose tissue is essential to improve visualization and facilitate precise preparation of the vessel. Since the IVC has a very thin wall and is prone to rupture, atraumatic handling is crucial to avoid vessel damage and ensure successful graft harvesting.

#### Basic Protocol 2: Preparation of the common carotid artery

Successful carotid artery preparation depends on careful and atraumatic vessel exposure while maintaining the integrity of the surrounding structures. During mobilization, special care must be taken to avoid injury or compression of the vagus nerve. Silk sutures should be used instead of Prolene, as Prolene is too stiff and can slip during fixation. Correct placement of the aneurysm clip on both the artery and the cuff handle is essential to prevent the arterial stump from retracting into the cuff. Flushing the arterial lumen with heparin solution is required to prevent clot formation and minimize the risk of embolic complications.

#### Basic Protocol 3: IVC graft interposition

Successful venous graft implantation requires careful positioning and atraumatic fixation of the vein between the carotid artery stumps. Excessive traction on the artery must be avoided, as too much tension can cause vessel tearing, before even placing the vein segment. The venous graft should be positioned with the diaphragmatic end first, as this allows easier handling and placement. Repeated flushing with heparin solution is essential to prevent clot formation and maintain vessel patency. Laser Doppler imaging should only be performed when the mouse is adequately warmed, as body temperature significantly affects blood flow measurements.

### Troubleshooting

Table 2 provides troubleshooting recommendations for common technical issues encountered during vein graft transplantation, including rupture of the thoracic inferior vena cava, arterial stump slippage into the cuff, arterial stump rupture, difficulties in graft placement, and absent graft perfusion on laser Doppler imaging.

**Table 2:** Troubleshooting Guide for Interposition of the IVC Into the Common Carotid Artery

Problem	Possible cause	Solution
Rupture of the thoracic inferior vena cava during harvesting	Excessive traction on the adventitial fat	Carefully mobilize the adventitial fat using forceps and microsurgical scissors, starting at the diaphragm
The caudal end of the arterial stump slides into the cuff	The clip clamps only the cuff instead of both the artery and the cuff	Reposition the clip to securely clamp both the artery and the cuff; dry the vessel and re‐clamp if necessary
Rupture of the arterial stumps while approximating the vessel	Excessive traction on the caudal arterial stump	Move the cranial arterial stump closer to the caudal stump to reduce tension
Difficulty pulling the vein graft over the caudal cuff	Incorrect orientation of the vein graft	Use the diaphragmatic end first, as its narrower diameter makes placement more challenging; the wider cardiac end is easier to mount onto the second cuff
The graft does not appear patent on laser Doppler imaging	The mouse's body temperature is too low; the anesthesia is too deep	Warm the mouse and, if necessary, reduce the depth of anesthesia

### Understanding Results

If protocols are performed correctly, users should observe the following.

In situ: After restoration of blood flow, successful graft function can be confirmed by visible pulsation and perfusion (Fig. 3F).

Laser Doppler imaging: In animals with successful surgery, a higher number of perfusion units should be detected in the operated artery. These values do not directly represent blood flow, but rather the amount of blood volume present within the measured vascular segment at the time of imaging. Since the venous graft has greater compliance and expands (dilates) under arterial pressure, it can accommodate a larger blood volume compared with a native artery, resulting in increased perfusion values (Fig. 4).

### Time Considerations

With prior microsurgical experience, Basic Protocol 1 requires 10 min while Basic Protocols 2 and 3 require together ∼50 min.

### Discussion

We present a simplified and reproducible mouse model for venous graft interposition using the inferior vena cava implanted into the common carotid artery. The method reduces technical complexity while preserving key hemodynamic features relevant to venous graft arterialization.

Venous graft failure remains a major limitation of coronary artery bypass grafting, and the mechanisms governing graft adaptation and long‐term patency are not fully understood. This model enables the investigation of arterial and venous grafts under standardized conditions, with a predefined cuff diameter ensuring highly comparable hemodynamic stress across animals.

The procedure proved to be feasible and safe, with a short operative time, absence of postoperative complications, and 100% survival during follow‐up. Regular laser Doppler imaging confirmed graft patency and blood flow in all animals, indicating successful graft function. However, laser Doppler imaging has several limitations. Perfusion measurements are sensitive to body temperature, anesthesia depth, and probe positioning, which may affect the recorded signal. Inter‐animal variability can further influence the measurements. In addition, laser Doppler provides a relative measure of perfusion and cannot directly quantify volumetric blood flow. Therefore, the results should be interpreted primarily as an assessment of relative graft perfusion and patency. Direct assessment of graft blood flow would require an imaging technique such as Doppler ultrasound; however, in our model, reliable ultrasound detection of the graft is challenging due to postoperative scar tissue surrounding the graft, which limits its visualization.

Although the model does not fully replicate the coronary circulation, it provides a robust and accessible platform for mechanistic and interventional studies of venous graft remodeling and patency in vivo.

### Author Contributions

**Vanessa Heim**: Conceptualization; data curation; formal analysis; methodology; software; visualization; writing—original draft. **Maria Ioannou‐Nikolaidou**: Data curation; investigation; methodology. **Anh‐Vu Nguyen**: Investigation; resources. **Lynn Muller**: Investigation. **Veronika Niedrist**: Investigation. **Jakob Hirsch**: Investigation. **Jonas Eder**: Investigation. **Dominik Hau**: Investigation. **Leo Winter‐Pölzl**: Conceptualization; project administration; supervision; writing—review and editing. **Clemens Engler**: Investigation. **Ronja Lohmann**: Investigation. **Felix Nägele**: Investigation. **Gerald Brandacher**: Writing—review and editing. **Stefan Schneeberger**: Writing—review and editing. **Nikolaos Bonaros**: Funding acquisition. **Michael Grimm**: Funding acquisition. **Can Gollmann‐Tepeköylü**: Funding acquisition; supervision; validation; writing—review and editing. **Michael Graber**: Data curation; formal analysis; funding acquisition; project administration; resources; supervision; validation; writing—review and editing.

### Conflict of Interest

The authors declare no conflicts of interest.

## Supporting information




*Transcription of Video 1*.

## Data Availability

The data, tools, and materials (or their source) that support the protocols are available from the corresponding author upon reasonable request.
