# Supplementary material for: Murine Model of Venous Bypass Grafting to Study Determinants of Graft Patency
Source: Curr Protoc. 2026 Sep 23;6(9):e70466. doi: 10.1002/cpz1.70466 (PMC13600260; doi:10.1002/cpz1.70466)
Supplement: Supplementary file 1 [file CPZ1-6-0-s001.docx]

**Transcription of video**

Perform a right median cervical incision from the manubrium sterni to the ear.

Carefully mobilize the sternocleidomastoid muscle, lift the muscle gently using a 4-0 Prolene suture for better exposure of the underlying

Remove the internal jugular vein around the common carotid artery and mobilize the vagus nerve. CAVE: make sure to not compress the artery and nerve.

Mobilize the common carotid artery from the bifurcation down to the brachiocephalic trunk

Ligate the artery at two points with an 8-0 silk suture while carefully avoiding injury to the nerve before transecting the artery**.** CAVE: use silk sutures instead of Prolene, as Prolene is too stiff and tends to slip.

Place the cuff over the caudal end of the artery using the long silk suture to guide it in position

Clamp the artery and the cuff exactly on the handle with an aneurysm clip as far distally as possible to secure the vessel and cuff in place. CAVE: It is very important that the artery and cuff handle are clamped with the clip, otherwise the stump of the artery will slip into the cuff.

Perform the same procedure on the cranial end of the artery. When done, use a 3-0 Vicryl suture and carefully wrap in around the clips

Cut the caudal artery directly behind the ligature, the arterial stump should be equally long as the cuff body, rinse the lumen with 100 IU/ml heparin solution. CAVE: It is important to flush the stump with heparin to prevent blood clots.

Take the stump of the artery and carefully evert it over the cuff body, use a cotton swab to hold the stump in position

Pull the double ligation loop around the everted artery and cuff, tie it tightly, and secure with a third knot

Rinse again with heparin solution.

Perform the same procedure with the cranial arterial stump

Use the already placed 3-0 Vicryl suture around the clips, crisscross and pull the sutures to bring the two ends of the arteries closer together**.** CAVE: Do not tighten too much, otherwise the carotid artery will be damaged.

Place the vein segment beside the artery stumps.

Rinse the cranial stump again with a heparin solution before placing the vein.

Position the venous end from the diaphragmatic side over the cranial arterial stump

Pull the double ligation loop around and knot it tightly, secure with a third knot, ensure that the vein is completely and evenly tightened

Position the last double ligation loop around the vein

Rinse the caudal arterial stump with heparin solution and pull the vein over, use a cotton swab to hold the vein in position

Pull the last double loop ligation over the vein and secure with a third knot

Complete full systemic heparinization (130 IU heparin) via IP injection before opening the clips.

After two minutes the cranial clip was removed first, then the caudal one

Pulsation and blood flow can be seen, if the surgery was successful

Remove all temporary sutures that were used to hold structures in place

.

Close the incision with a running suture using 4-0 Prolene
